# The Patient Centered Assessment Method (PCAM): integrating the social dimensions of health into primary care

**DOI:** 10.15256/joc.2015.5.35

**Published:** 2015-07-16

**Authors:** Rebekah Pratt, Carina Hibberd, Isobel M. Cameron, Margaret Maxwell

**Affiliations:** ^1^Department of Family Medicine and Community Health, University of Minnesota, Minneapolis, MN, USA; ^2^School of Nursing, Midwifery and Health, University of Stirling, Stirling, UK; ^3^Psychiatry Group, Division Institute of Applied Medicine, Health and Wellbeing, University of Aberdeen, Clinical Research Centre, Royal Cornhill Hospital, Aberdeen, UK

**Keywords:** Patient Centered Assessment Method (PCAM), comorbidity, multimorbidity, social dimensions of health, prevention, patient complexity

## Abstract

**Background:**

Social dimensions of health are known to contribute to what is often termed “patient complexity,” which is particularly common among patients with multimorbidity. Health-care professionals require tools to help them identify and manage these aspects of patient needs.

**Objectives:**

To examine: (i) the Patient Centered Assessment Method (PCAM), a tool for assessing patient complexity in ways that are sensitive to the biopsychosocial dimensions of health, in primary care settings in Scotland; (ii) the impact of the PCAM on referral patterns and its perceived value; and (iii) the PCAM’s perceived applicability for use in a complex patient population.

**Design:**

Two studies are described: (i) a mixed-methods prospective cohort study of the implementation of the PCAM in primary care clinics; and (ii) a qualitative exploratory study that evaluated the value of the PCAM in a complex patient population.

**Results:**

Use of the PCAM did not impact patient satisfaction or perception of practitioners’ empathy, but it did increase both the number of onward referrals per referred patient (9–12%) and the proportion of referrals to non-medical services addressing psychological, social, and lifestyle needs. Nurses valued the PCAM, particularly its ability to help them address psychological and social domains of patients’ lives, and found it to be highly relevant for use in populations with known high complexity.

**Conclusions:**

The PCAM represents a feasible approach for assessing patient needs with consideration to the social dimensions of health, and allows practitioners to refer patients to a broader range of services to address patient complexity.

## Introduction

The presence of multiple chronic conditions is a growing reality for many patients, and is shaping how care is delivered and assessed. Much of the research on the experience of illness has focused on single conditions [1], yet, with a growing realization of the importance of multimorbidity, researchers and health-care providers alike must explore new conceptual models and measures to better describe and address multimorbidity in relation to patient experiences and health outcomes. The conceptual models used to consider how to address illness have been dominated by the Chronic Care Model (CCM) [2]; however, this model has been critiqued for failing to articulate in greater detail the potential community resources aspect of the model [3, 4]. While the CCM is a comprehensive and well-tested model, it suggests that the “informed, activated patient” somehow sits outside of the broader social influences of the community and health system. Research on multimorbidity demonstrates a compelling link with broader social dimensions of health, and indicates a need to find ways to make these social determinants and patient experiences more central to the conceptual model [5]. The Innovative Care for Chronic Conditions (ICCC) framework further responds to the limitations of the CCM in an international context, and considers the broader policy and community context of care [6, 7].

It is being increasingly recognized that the social determinants of health that characterize socioeconomic disadvantage lead to a complex interplay of biological, psychological, and social factors that contribute to poor health outcomes [8, 9]. Often in primary care, the impact of these various characteristics is conceptualized as “patient complexity” [10]. Attempts to identify and address the social dimensions of health have led to a renewed interest in how the context of patients’ lives, as well as broader social and economic conditions, influence the experience of illness and the success of treatment for many conditions [9]. It is also well-established that patients living in more deprived circumstances are more likely to experience multimorbidity [5, 11], which further highlights the need to take a comprehensive view of a patient’s life circumstances as part of providing effective care.

In Scotland, the impact of the social determinants of health is particularly evident in the area of heart disease. While deaths from coronary heart disease are decreasing overall, they are decreasing less quickly for those in the most economically deprived population; in addition, life expectancy appears to be decreasing in some of the most deprived areas in Scotland [11]. One of the key initiatives that arose from these concerns was “Keep Well,” a Scottish government initiative to conduct anticipatory health checks targeting cardiovascular disease, along with diabetes risk identification and reduction, in areas of high socioeconomic disadvantage. Keep Well sought to consider cardiovascular risk in a context that is responsive to the social dimensions of health. Heart disease, in general, is also associated disproportionately with those in both mentally [12, 13] and physically [14, 15] ill health, and more so for patients from low socioeconomic groups [16, 17] who may also be less likely to engage in self-care [16]. Poor mental well-being is also a risk factor for coronary heart disease [18–20] and potentially for type 2 diabetes [21]. For this reason, after its initial implementation to assess cardiovascular disease and diabetes risk factors, the Keep Well program subsequently sought to integrate mental-health screening into its anticipatory health checks. 

These challenges to considering mental health in a context that was sensitive to the complex interplay of mental and physical health (both of which were influenced by the social dimensions of health) highlighted the need to conduct a comprehensive assessment that would consider all of these aspects of a patient’s needs. The Patient Centered Assessment Method (PCAM) was developed as a Keep Well anticipatory health-check screening tool [22] to integrate the social dimensions of health into primary care practice.

The PCAM has its origin in the Minnesota Complexity Assessment Method (MCAM) [23], which was created to bring a broad range of aspects of health into patient assessments, including physical health, mental health, social support, social needs, health literacy, and engagement with the appropriate services. The MCAM was derived from “INTERMED,” a screening tool that integrated biopsychosocial aspects of the relationship between the patient and the health-care system to reflect “case complexity,” and has been used in secondary care [24, 25]. The purpose of the MCAM was to provide a practical, but systematic, vocabulary and action-based evaluation system that could be applied in a primary care outpatient setting. The MCAM has also been used to help improve interdisciplinary teamwork in educational settings [26]. The PCAM is orientated to patient-centered assessment, and is suitable for use with patients who may have comorbid conditions or multimorbidity. The focus of the assessment is to incorporate the patient’s broader life context in a way that accommodates the patient’s experience of their health.

While the conceptual basis for assessing complexity has been established via the INTERMED and MCAM, further adaptation and validation was required for use in a UK health context and for use in Keep Well health-screening activities to ensure the USA-developed tool could be adapted for use in the UK [22]. This led to the development of the PCAM as an adapted version of the MCAM. The name was changed following feedback from nurses participating in this study, indicating a preference to emphasize the patient centeredness of the assessment, rather than focus on the word complexity. The PCAM was developed to provide a comprehensive assessment of biopsychosocial needs, and to encourage practitioners to identify action in relation to identified needs based on the severity and level of urgency surrounding those needs. It was intended that the PCAM would help to identify biopsychosocial complexities in a manner that facilitated referral to the appropriate medical, lifestyle, psychological, social, and self-help services in a more effective way. In settings where social dimensions of health cannot be addressed in the primary care environment, the goal of PCAM is to encourage referral outside of the primary care clinic to those who can address the social dimensions of health identified by the PCAM. Or premise is, in doing so, patients can be provided with opportunities to address aspects of their life which impact their health, even if they are not issues that can be addressed by the primary care provider. The PCAM can be used by a range of primary care providers, including GPs or nurses. It may also be used by a team of providers to assist with communication for team-based approaches to care. The PCAM was not developed for cardiovascular disease or diabetes population exclusively, but rather was tested in this group for the feasibility of use in primary care with these commonly occurring conditions, and was deemed suitable for use in this study because of its focus on the social dimensions of health.

The primary aim of the research was to develop and establish face validity of the professional version of the PCAM, specifically in relation to its ability to identify mental-health-related needs. Secondary aims of the research were to commence the process of some preliminary external validity testing of the PCAM (reported elsewhere [27]), to establish how best to integrate the PCAM into the existing Keep Well health checks, and to assess the implementation of the PCAM (including the impact on perceived provider empathy and patient satisfaction). This report also describes the PCAM’s perceived applicability and acceptability among a small group of professionals working with populations with highly complex needs. To do this, we focused on measures of how the use of the PCAM impacted the consultation itself, by examining the impact on patient satisfaction, patient experience, and referral patterns. The ability of the PCAM to determine social dimensions of health is reported elsewhere [22], although fully establishing this remains an ongoing research endeavor.

## Methods

This research was conducted in two separate studies. Study 1 was a mixed-method study exploring patient-reported satisfaction and perceived provider empathy and nurse-initiated onward referral for two separate cohorts of patients (pre- and post-implementation of the PCAM), as well as collecting qualitative data on nurses’ experiences in implementing the PCAM. This study was conducted within Keep Well clinics in two National Health Service Boards. Keep Well was a screening program, which was hosted by local primary care clinics. Nurses from Keep Well would send invitations to local patients to attend a screening appointment in addition to any usual care they received from their local health-care provider. Usual care would typically involve patient management by a GP, with support of the practice nurse. The Keep Well screening appointments were an additional service targeting patients considered at risk of cardiovascular disease on the basis of age and geographical location. Study 2 was a qualitative exploratory study asking health-care professionals working in primary care practices serving a known highly complex population (homeless, refugee, and travelling communities) to evaluate the applicability and acceptability of the PCAM for professionals serving that population. Qualitative methods were used as an essential method for collecting data about experience and perceptions of the tool. Qualitative methods were used to collect data from staff only in the second study.

### Study 1

A total of ten nurses, five from Site 1 and five from Site 2, agreed to take part in the implementation study and data collection. As part of the general Keep Well training, these nurses were all trained in mental-health awareness and identification of mental-health problems, with particular emphasis on the impact of social dimensions of health. Further brief training was provided on the use of the PCAM itself, emphasizing how to integrate the assessment into the clinical encounter, how to respond to the information collected in the PCAM, and how they would respond to clinical case examples.

Baseline data (pre-PCAM implementation) were collected to assess patient satisfaction with the Keep Well anticipatory health checks as well as to record the actions (including non-medical referrals) initiated by Keep Well nurses. Baseline data were collected by recruiting up to 50 consecutive patients (prorated for part-time nurses) attending the anticipatory health checks of the study nurses, and asking them to complete the Client Satisfaction Questionnaire (CSQ) [28] and the Consultation and Relational Empathy measure (CARE) [29] after their health check. These could be completed at the clinic and left in a sealed box or taken home and returned by post at a later date. Nurse and patient questionnaires were linked using a study identification code, but patients remained anonymous. Where possible, a researcher was present in the clinic to explain the study, answer questions, and assist with questionnaire completion.

Following baseline data collection, nurses began to implement the PCAM in their practices. Follow-up data (post-PCAM implementation) were also collected, which involved repeating the same data collection as at baseline, but including the PCAM form (completed by nurses), for different patients from the baseline data. The PCAM is an 11-item assessment covering three domains (health and well-being; social environment; health literacy, and communication) assessed on their level of severity and urgency. This assessment is then accompanied by a section to record the actions that will be taken in relation to these needs. Across each domain, item questions ascertain the level of need across a four-point scale, with 1 indicating no needs or issues relating to the item, through to 4, indicating urgent or serious needs or issues. 

Finally, semi-structured qualitative interviews were conducted with all participating nurses to evaluate the process of implementation, and to identify any perceived advantages or difficulties in using the PCAM. Nurses were recruited to take part in interviews by the research team, and conducted in person, recorded, and transcribed the interviews for analysis. 

### Study 2

Staff nurses in one “access practice” serving homeless, refugee, and travelling populations took part in a qualitative exploratory study of the applicability, acceptability, and feasibility of using the PCAM in this population group. One focus group (*n*=6) was conducted with staff nurses prior to the use of the PCAM to obtain their views about the tool, following this, three nurses piloted the use of the PCAM with 18 patients. The patients were selected by the nurses, and were identified on the basis that the nurses perceived it would be a useful learning case, or useful to apply the PCAM to those particular patients. The nurses then discussed their experience with the PCAM in a follow-up focus group (*n*=6, 5 of whom participated in the first focus group).

### Data analysis

For Study 1, baseline data (CSQ, CARE) were collected on 286 patients, and post PCAM implementation, further data (CSQ, CARE, PCAM) were collected on a new cohort of 243 patients. Table 1 shows the number of patient-completed questionnaires and patient demographics for both the baseline and follow-up populations. Baseline and follow-up scores for the CSQ and CARE measures were compared using the Mann–Whitney U test, because the data were categorical and skewed towards most satisfaction (CSQ) or highest quality (CARE). A *p*-value of <0.01 was considered statistically significant. In addition, patterns of patient referrals were examined for pre- and post-PCAM implementation differences.

Qualitative data obtained from nurses in both studies were analyzed using a social-constructivist version of the grounded theory [30], which allowed for the identification of themes and subthemes. All interview and focus-group data were audio recorded and fully transcribed. NVivo9 (QSR International) was used to help facilitate the process of analysis. All emerging themes were discussed and reviewed by three members of the research team, allowing for areas of difference to be explored and reviewed until a consensus was reached about the emerging analysis. A small number of quotations have been included in this report for the purpose of illustrating our findings.

### Study participation and completion rates

For Study 1, patients were only included in the analysis if both nurse- and patient-completed questionnaires were available and if the nurse participated in both baseline and follow-up data collection. Thus, we were able to use 74% (286/389) of data collected at baseline and 97% (243/251) of the data collected at follow-up. The loss of data was largely due to the loss of three nurses from the study, one of whom withdrew just as post-implementation (follow-up) data collection was beginning.

### Ethical approval

Approval for Study 1 was granted from the East of Scotland Research Ethics Committee (REC 10/S0501/44), together with R&D approval (NRS10/GH13). The East of Scotland Research Ethics Service decided that Study 2 represented service development (given that the protocol did not affect patient care) and that Research Ethics Committee approval was therefore not required. Written informed consent was obtained by researchers from participants for Study 1. A member of the research team, who was not part of the clinic staff, was present onsite, and conducted the consent process with patients, making clear that their participation would not impact their clinical care and their data would remain confidential from their health-care provider. For Study 2, written consent was not obtained based on the Research Ethics Services’ categorization of the project. In relation to qualitative data collected, all participants were assured that their anonymity would be maintained. All data collected were assigned codes and identifying information was removed. The research team members were the only people to hold the key to the codes for participants. They did not have dual clinical roles, and only performed research tasks.

## Results

### Study 1

#### Description of patient sample

No statistical differences were observed in the age of the patient populations between Sites 1 and 2 at baseline or follow-up (Table 1; *p*>0.05 in both cases). However, whereas the mean age at Site 2 was equivalent in the baseline and follow-up populations, at Site 1, the baseline population was significantly older than the follow-up population [unequal variance *t*-test, *t*=5.362, degrees of freedom (df)=262.002, *p*<0.001, 99% confidence interval 1.743 to 5.012]. In terms of gender, there were proportionately more males at Site 1 than at Site 2 at baseline [Chi-square (c^2^)=28.081, df=1, *p*<0.01], but more males at Site 2 than at Site 1 at follow-up (c^2^=13.5, df=1, *p*<0.01). In addition, at Site 1, the proportion of males was significantly higher in the baseline population than in the follow-up population (c^2^=90.264, df=1, *p*<0.01), whereas at Site 2, the proportions of males in the baseline and follow-up populations were not significantly different (c^2^=91.125, df=1, *p*<0.01). 

#### PCAM form responses for the follow-up population

Table 2 shows the distribution of scores across the PCAM for the follow-up population sample, including completion rates for each PCAM item. Completion rates for each item exceeded 90%, except for the Action item, which had a completion rate of 88%. The distribution of responses for the items that addressed the social environment domain largely reflected the pattern of responses obtained for the health and well-being domain. For the two health literacy questions, over 90% of responses were to the Answer 1 option, relating to reasonable patient understanding. Answer 4, indicating the greatest extreme of need, was infrequently endorsed for most items, but its inclusion remains appropriate to allow scope for indicating extreme need, even if rarely required.

#### Patient experience

A measurement of patient satisfaction (CSQ) was taken at both baseline (pre-PCAM implementation) and follow-up (post-PCAM implementation). Patient satisfaction item scores did not differ significantly between the baseline and follow-up populations at each site, with the exception of question 8 (“If you were to seek help again, would you come back to our service?”), at Site 2 (Table 3). No significant differences were seen in the data between phases at either site. It is possible that the data reflect a ceiling effect (social desirability bias), which can occur with satisfaction questionnaires. Non-recorded discussion with patients (by researchers in the waiting area during completion of patient questionnaires) revealed that some of the CSQ questions were less applicable to a Keep Well health check than, for example, a patient-initiated general practitioner appointment for routine care. These informal discussions also indicated a high level of satisfaction among baseline and follow-up cohort patients with the Keep Well service, indicating that the results obtained may have been due to real satisfaction and not just social desirability bias. Therefore, high general satisfaction at baseline may have precluded the detection of improved ratings. Analysis at the individual nurse level did not reveal any pattern of significant differences.

A measure of practitioner empathy (CARE) was also collected at baseline and follow-up. No significant differences were observed in the empathy item scores between the baseline and follow-up populations at each site (Table 4). As for the CSQ, it is possible that a ceiling effect also impacted this measure. Analysis at the individual nurse level (in the four nurses who completed a minimum of 20 questionnaires) did not reveal any pattern of significant differences.

The lack of significant changes in the CSQ and CARE measures between the baseline and follow-up populations suggest that satisfaction and empathic communication did not increase with the use of the PCAM, and neither did it decrease, indicating that the PCAM did not have any negative impact on satisfaction and empathy either.

#### Impact of the PCAM on referral patterns

This study sought to identify whether the numbers of referrals or the patterns of referrals to other (non-medical) services changed as a result of using the PCAM. Following the introduction of the PCAM, there was a decrease in referrals for medical services and an increase in psychological, social, and lifestyle referrals. At Site 1, the proportion of patients receiving a referral decreased by 18% (absolute difference; 67% to 49%) in the follow-up (post-PCAM implementation) population compared with the baseline population. However, recorded referrals made per referred patient increased by 12% (1.16 to 1.30) in the follow-up population compared with the baseline population at this site. At Site 2, the proportions of patients receiving a referral decreased by 14% in the follow-up population compared with the baseline population, and the number of referrals made per referred patient increased by 9%. These observed differences could be due to the use of the PCAM, to differences in the population, or to differences in recording. For both sites, medical referrals as a proportion of referrals decreased, whereas the proportion of non-medical (psychological, social, and lifestyle) referrals increased, when the follow-up population was compared with the baseline population.

Although these data indicate that the use of the PCAM was associated with a broader range of referrals being made in order to address patient needs, conclusions about the impact of the PCAM upon the number of referrals being made could not be drawn from this study because of the differences in the baseline and follow-up populations. 

#### Feasibility and impact of implementing the PCAM

Analyses of nurse interview responses indicated high levels of nurse practitioner support for the PCAM. The PCAM was perceived to enhance professional practice, to promote their ability to authentically engage in holistic assessment, to offer benefits to patient care, and to support the willingness of patients to open up about problems when given the opportunity.

Nurses were highly supportive of the value of using the PCAM and reported little difficulty in using it. They reported that, in general, it took completion of 10–15 PCAM assessments with patients for them to develop confidence in its use. For some nurses, it led to new types of interactions with patients as they explored psychological and social domains of the patients’ lives, whereas for others, it provided a mechanism to record some of the in-depth conversations they were already having with patients. In particular, nurses valued the “action”-oriented requirement of the PCAM.

In addition, many nurses indicated that they perceived the PCAM as augmenting their existing practice and perhaps raising their awareness about addressing mental well-being and the surrounding social issues that can impact mental health. *“I think that it helped, I suppose it’s helped me to think or to give a bit more focus to these other issues that potentially might be around…it’s improved my awareness and improved my focus during the assessment.” (Site 2).*

The PCAM aims to improve psychosocial assessment or holistic assessment of needs, and this approach was very much endorsed by the nurses. It encouraged them to move beyond a focus on physical concerns. Indeed, the PCAM was seen as the antithesis of the “tick-box” assessment. *“What I mean by that is a health check question is how many units of alcohol you drink a week, that could be just a tick box type of thing, but I think the PCAM adds to it in a sense where you’re actually trying to get the meaning of what that means to somebody and then picking it up from there…it’s helped me at a personal level about the aches and pains and the abuse and other things that often go on for people but I suppose [are] not asked about.” (Site 2).*

In terms of integration into the Keep Well health check, all nurses participating in the study agreed that the PCAM could be integrated into a health check. For brief health checks (20–30 minutes), 10–15 additional minutes were needed to enable PCAM form completion. For longer health checks (50 minutes), the completion of the PCAM form took only a few minutes longer, since the ongoing conversation within the health check had already elicited much of the information required. Nurses noted that they expected that the process could be made more efficient, such that, with practice development and increased nurses’ experience, a health check that included completion of the PCAM form could be done within 30 minutes. The nurses had no major suggestions for any changes to the PCAM; indeed, they endorsed all the domains within the tool as being necessary and comprehensive in terms of the context of their patients’ lives. The nurses reported that they valued the tool in identifying issues for patients and helping to point them towards the appropriate medical or social support.

A concern at the outset of the study was that using the PCAM would open a “can of worms” in terms of serious issues being expressed by patients and that the nurses would have a “lack of options” for dealing with the problems that might be identified. In general, use of the PCAM did not generate either a volume or type of responses that could not be addressed. Relatively few patients reported problems of significant severity or urgency, and where problems were uncovered, most staff felt that options were available to refer patients to. *“I think we had the support network set up already so then I didn’t feel I kind of came away from that no[t] being able to follow through with the support after bringing the subject up. Otherwise a lot of the patients I’ve talked [through] the PCAM with, it’s been very straightforward.” (Site 1).*

Overall, the PCAM was perceived as having a positive impact on patients and the services they received: nurses felt that patients would get a more thorough health check and the offer of some help for any identified mental-health and well-being problems. In the majority of cases, the nurses were quite surprised at how patients had welcomed the opportunity to discuss problems. *“…the patient is very much more open and willing to talk about things than I thought they would be.” (Site 1).*

### Study 2

To explore the feasibility of the use of the PCAM in a highly complex patient population, we examined its use in a primary care “access” practice serving homeless, refugee, and travelling communities. Despite the high biopsychosocial complexity of these client groups, focus-group findings indicated that the practice nurses felt that tools or assessments that had been tested sufficiently for use with these population groups were lacking. This led to some concern that standardized measures might not always be appropriate for working with these populations. *“Because as I say I mean we’re using potentially in these groups homeless and Gypsy Travellers, people who are constantly living in this “no light at the end of the tunnel existence” and we’re using a risk assessment tool….I think there’s a lot of good evidence around it but it’s an apple and our people are oranges.” (Nurse “Access Practice”).*

In the follow-up focus group conducted after piloting the PCAM with a small cohort of these patients, the PCAM format was favorably received by the nurses, and the domains were seen to be relevant. Nurses described that, in their experience with these populations, client mistrust of paperwork or assessments often exists, highlighting the importance of introducing the PCAM as a collaborative tool that was helping to inform the consultation. In this sense, the assessment could be seen as a tool to help facilitate conversation, or complement a motivational-interviewing style. *“But the other idea is to use that, like [nurse] was saying to develop it, using a prompt, a prompt for conversation and I really like that idea and you’ve got your tool which could be that, yeah I like that. But I really like the idea of having something a bit more elaborated that’s just a working tool, or indeed for the patient if they want to take it away with them, some will.” (Nurse “Access Practice”).*

Further, focus-group participants indicated that they felt that the PCAM was more appropriate than other standard assessments for these pilot highly complex populations, and was both acceptable and feasible for use with these patients, particularly in the context of facilitating motivational interviewing-based consultations. PCAM was seen to have good potential to support that relationship building, especially if used as a framework or tool that could be completed collaboratively with the patient. Lastly, the nurses perceived the PCAM to be well-received by the patients in the pilot study.

## Discussion

The current study has tested the implementation of an assessment tool that can guide a discussion of biopsychosocial issues for nurses delivering anticipatory care health checks that is sensitive and responsive to the social dimensions of health. Nurses, who dealt with patients with a range of complex issues, including long-term conditions, reported that they valued the tool in identifying issues for patients and helping to point them towards appropriate medical or social support. In terms of integration into the Keep Well health check, all nurses participating in the study agreed that the PCAM could be integrated into a health check. There were no major suggestions for any changes to the PCAM; indeed, there was endorsement of all the domains within the tool as being necessary and comprehensive to the context of their patients’ lives. The study demonstrated that introducing such an assessment into practitioner workflow was possible, and valued. Achieving this required support for the nurses to use such an approach, and the development of links with agencies or resources, which could serve as sources of referral or information relating to these social dimensions of health. 

There are challenges in considering how a tool such as the PCAM can be integrated into usual care for patients. In the UK, recent primary care-led assessments of mental-health problems in people living with chronic or long-term conditions did not have the intended impact [31–33]. This is likely due to the limited experience and lack of confidence of nurses (who conducted most depression screening) in mental health. Furthermore, in these assessments, little or no attention is paid to the social problems that might contribute to poor physical and mental well-being. Indeed, the use of population-level mental-health screening in deprived communities has a number of inherent challenges, such as the identification of potential problems of a scale that would outstrip the available local services or the over-medicalization of distress (the latter of which can not only be stigmatizing for patients, but may also potentially supplant more integrated holistic approaches that support prevention and health-promotion approaches) [34–37].

Improvement models from the USA (such as those used by Kaiser Permanente, Pfizer, and Evercare) have been promoted to enhance the care of people with long-term conditions. These models are based on having a care manager, who has a key role in coordinating services from other health and social care providers [37]. However, in UK primary care, this ideal has moved little beyond nurses conducting annual health checks (as encouraged under the Quality and Outcomes Framework) that meet the requirements of the general practitioners payment system. The Royal College of General Practitioners in the UK promotes care planning [38], but acknowledges that co-existing mental and social circumstances may prevent such approaches. For example, the Royal College’s response to Quality and Outcomes Framework indicators for depression noted that *“A holistic assessment should be part of the routine management of any patient with a long term condition.”* However, few validated tools for such assessment exist, especially for use by nurses or care managers. The development of interventions for primary care that encourage holistic assessment and action to address complex health and social needs is urgently required. The benefits of doing so include increased referrals to other (non-medical) services to address comorbid psychosocial issues.

### Study limitations

This study has focused on developing the PCAM for use within Keep Well (and similar) anticipatory health checks provided by nurses. As such, the potential to extend its use to other primary care settings and professionals has not been explored. This study also attempted to measure the outcomes or impact of using the PCAM during its early development and implementation. It was clear that many nurses took some time to familiarize themselves with completing the tool, to become “comfortable” and “confident” in its use. As a result, measuring “impact” may have been undertaken too early and the study could have benefitted from more time for nurses to become more familiar with its use before collecting information on changes in referral patterns. Additionally, the PCAM was only tested in a certain population, i.e. Scottish patients identified as being at risk of coronary heart disease in low socio-economic areas, and the use of two separate cohorts for pre-and post-PCAM evaluation potentially make it difficult to interpret the findings.

## Conclusions

The PCAM can be a valuable tool in encouraging holistic assessment of patients’ biopsychosocial needs and addressing patient complexity. With training and practice, it can be easily integrated into nurse-conducted health checks. The potential of the PCAM to be used in other primary care settings and health systems (e.g. in the USA) should be explored. The impact of the PCAM on longer-term patient outcomes should also be explored. Combined with the use of motivational interviewing, use of the PCAM might lead to better patient involvement in disclosing problems and engaging in referrals to address social complexity.

## Figures and Tables

**Table 1:** Patient demographics and number of questionnaires completed.

Patient demographics	Cohort	Site 1	Site 2
Completed questionnaires, N	Baseline	119	167
	Follow-up	108	135
Age in years, mean (SD)	Baseline	54 (6)	52 (12)
	Follow-up	50 (5)	52 (11)
Gender, N male (%)	Baseline	76 (64)	102 (61)
	Follow-up	41 (38)	75 (56)

SD, standard deviation.

**Table 2:** Patient Centered Assessment Method (PCAM) item answers and completion rates in follow-up population (*n*=243), Study 1.

Item	Answer, N (%)				Item completed, N (%)
	1	2	3	4	
Health and well-being					
Physical health needs	129 (54)	74 (31)	36 (15)	0	239 (98)
Physical health problems impacting on mental health	148 (61)	62 (26)	29 (12)	2 (<1)	241 (99)
Lifestyle impacting on mental health	156 (65)	64 (27)	19 (8)	0	239 (98)
Other concerns impacting on mental health	154 (65)	59 (25)	25 (10)	0	238 (98)
Social environment					
Home environment: impact on health and safety	191 (79)	44 (18)	6 (3)	0	241 (99)
Daily activities	166 (70)	47 (20)	23 (9)	3 (1)	239 (98)
Social network	146 (60)	62 (26)	32 (13)	1 (<1)	241 (99)
Financial resources	155 (64)	71 (30)	12 (5)	2 (1)	240 (99)
Health literacy and communication					
Health literacy: understanding	217 (94)	14 (6)	0	0	231 (95)
Health literacy: communication	215 (92)	16 (7)	1 (<1)	0	232 (96)
Action	122 (57)	25 (12)	13 (6)	54 (25)	214 (88)

**Table 3:** Client Satisfaction Questionnaire (CSQ) item scores by site in baseline and follow-up populations, Study 1.

CSQ item	Site 1			Site 2		
	Median response (IQR)		*p*	Median response (IQR)		*p*
	Baseline	Follow-up		Baseline	Follow-up	
1. How would you rate the quality of care you have received?	4 (4–4)	4 (4–4)	0.054	4 (4–4)	4 (4–4)	0.321
2. Did you get the kind of service you wanted?	4 (4–4)	4 (4–4)	0.944	4 (4–4)	4 (3–4)	0.149
3. To what extent has the care provided by your Keep Well Checker met your needs?	4 (3–4)	4 (4–4)	0.118	4 (4–4)	4 (3–4)	0.075
4. If a friend were in need of similar help, would you recommend our service to him or her?	4 (4–4)	4 (4–4)	0.425	4 (4–4)	4 (4–4)	0.178
5. How satisfied are you with the amount of care you have received?	4 (4–4)	4 (4–4)	0.032	4 (4–4)	4 (4–4)	0.308
6. Have the services you received helped you to deal more effectively with your problems?	4 (3–4)	4 (3–4)	0.700	4 (3–4)	4 (3–4)	0.038
7. In an overall general sense, how satisfied are you with the service you have received?	4 (4–4)	4 (4–4)	0.763	4 (4–4)	4 (4–4)	0.769
8. If you were to seek help again, would you come back to our service?	4 (4–4)	4 (4–4)	0.875	4 (4–4)	4 (4–4)	0.004^*^

^*^p<0.01 level. IQR, interquartile range.

**Table 4:** Consultation and Relational Empathy (CARE) scores by site in baseline and follow-up populations, Study 1.

CARE item	Site 1			Site 2		
	Median response (IQR)		*p*	Median response (IQR)		*p*
	Baseline	Follow-up		Baseline	Follow-up	
1. Making you feel at ease	5 (4–5)	5 (5–5)	0.100	5 (4–5)	5 (4–5)	0.857
2. Letting you tell your story	5 (4–5)	5 (4–5)	0.070	5 (4–5)	5 (4–5)	0.099
3. Really listening	5 (4–5)	5 (5–5)	0.015	5 (4–5)	5 (5–5)	0.613
4. Being interested in you as a person	5 (4–5)	5 (5–5)	0.055	5 (4–5)	5 (4–5)	0.283
5. Fully understanding your concerns	5 (4–5)	5 (5–5)	0.087	5 (4–5)	5 (4–5)	0.677
6. Showing care and compassion	5 (4–5)	5 (5–5)	0.069	5 (4–5)	5 (4–5)	0.954
7. Being positive	5 (4–5)	5 (5–5)	0.236	5 (4–5)	5 (4–5)	0.704
8. Explaining things clearly	5 (4–5)	5 (5–5)	0.152	5 (4–5)	5 (4–5)	0.617
9. Helping you to take control	5 (4–5)	5 (5–5)	0.428	5 (4–5)	5 (4–5)	0.549
10. Making a plan of action with you	5 (4–5)	5 (4–5)	0.162	5 (4–5)	5 (4–5)	0.556

IQR, interquartile range.
